# Collaborations for Leadership in Applied Health Research and Care: lessons from the theory of communities of practice

**DOI:** 10.1186/1748-5908-6-64

**Published:** 2011-06-23

**Authors:** Roman Kislov, Gill Harvey, Kieran Walshe

**Affiliations:** 1 Manchester Business School, The University of Manchester, Booth Street West, Manchester, M15 6PB, UK

## Abstract

**Background:**

The paper combines the analytical and instrumental perspectives on communities of practice (CoPs) to reflect on potential challenges that may arise in the process of interprofessional and inter-organisational joint working within the Collaborations for Leaderships in Applied Health Research and Care (CLAHRCs)--partnerships between the universities and National Health Service (NHS) Trusts aimed at conducting applied health research and translating its findings into day-to-day clinical practice.

**Discussion:**

The paper discusses seminal theoretical literature on CoPs as well as previous empirical research on the role of these communities in healthcare collaboration, which is organised around the following three themes: knowledge sharing within and across CoPs, CoP formation and manageability, and identity building in CoPs. It argues that the multiprofessional and multi-agency nature of the CLAHRCs operating in the traditionally demarcated organisational landscape of the NHS may present formidable obstacles to knowledge sharing between various professional groupings, formation of a shared 'collaborative' identity, and the development of new communities within the CLAHRCs. To cross multiple boundaries between various professional and organisational communities and hence enable the flow of knowledge, the CLAHRCs will have to create an effective system of 'bridges' involving knowledge brokers, boundary objects, and cross-disciplinary interactions as well as address a number of issues related to professional and organisational identification.

**Summary:**

The CoP approach can complement traditional 'stage-of-change' theories used in the field of implementation research and provide a basis for designing theory-informed interventions and evaluations. It can help to illuminate multiple boundaries that exist between professional and organisational groups within the CLAHRCs and suggest ways of crossing those boundaries to enable knowledge transfer and organisational learning. Achieving the aims of the CLAHRCs and producing a sustainable change in the ways applied health research is conducted and implemented may be influenced by how effectively these organisations can navigate through the multiple CoPs involved and promote the development of new multiprofessional and multi-organisational communities united by shared practice and a shared sense of belonging--an assumption that needs to be explored by further empirical research.

## Introduction

Since being identified as a mechanism through which knowledge is held, transferred, and created, the communities of practice (CoP) approach has become increasingly influential within management research and practice [1]. Originally developed by Lave and Wenger [2] in a study of situated learning, the CoP theory is currently being used to analyse and facilitate knowledge sharing in a wide range of organisational environments, including, but not limited to, business sector, education, information technology (IT) and healthcare organisations. In the healthcare sector, CoPs have been argued to play a role in the generation of social, human, organisational, professional, and patient capital, thus being potentially useful for enhancing care, providing learning opportunities, analysing practice, problem-solving, sharing knowledge, and generating ideas [3].

This paper will use the CoP approach as a lens to look at interprofessional and inter-organisational joint working within the Collaborations for Leadership in Applied Health Research and Care (CLAHRCs)--partnerships between the universities and National Health Service (NHS) Trusts aimed at conducting applied health research and translating its findings into day-to-day clinical practice. It will briefly discuss some of the seminal theoretical CoP literature as well as previous empirical research on the role of CoPs in healthcare collaboration, using CoPs as a lens to reflect on potential challenges that the CLAHRCs will have to address in order to achieve their objectives. It will argue that the multiprofessional and multi-agency nature of the CLAHRCs operating in the traditionally demarcated organisational landscape of the NHS may present formidable obstacles to knowledge sharing between various professional groupings, effective identification with the Collaboration, and the formation of new multiprofessional communities within the CLAHRCs.

The paper will start with a brief discussion of the structure and purposes of the CLAHRCs and the main premises of the CoP approach. It will then explore the following three interrelated strands within the wider CoP literature: knowledge sharing across CoPs; CoP formation and manageability; and CoP identity building. The paper will conclude by discussing the advantages of applying the CoP theory to healthcare partnerships, summarising the key issues that need to be addressed by the CLAHRCs and similar organisations in order to achieve their aims, and reflecting on the implications for future research in this area.

## Background

### Collaborations for Leadership in Applied Health Research and Care

In 2008, another major experiment was launched in the English NHS: CLAHRCs were established across England. Each CLAHRC represents a collaborative partnership between one or more universities and their surrounding NHS organisations aiming to undertake high-quality, patient-centred applied health research and to support the translation of research evidence into clinical practice. In total, nine CLAHRCs were selected through an open competition out of twenty-two bids, with particular value being placed on research proposals targeted at chronic disease and public health interventions. Each of them will receive up to £10 m over five years from the National Institute for Health Research (NIHR), with additional matched funding to be secured from the participating NHS organisations to at least the same level as that provided by the NIHR [4,5].

The CLAHRCs are expected to enhance knowledge transfer between academic researchers and NHS staff and thus address the 'second gap in translation'--a gap in the translation of new medical interventions into everyday practice identified by Cooksey's *Review of UK Health Research Funding *[6]. They have three key interlinked functions: conducting high quality applied health research; implementing the findings from research in clinical practice; and increasing the capacity of NHS organisations to engage with and apply research. It should also be noted that the CLAHRCs are situationally placed, with their agendas being determined by the partnering organisations and tailored to the healthcare needs in their respective geographical areas.

The CLAHRCs can be considered as a somewhat experimental approach designed to further our understanding of large-scale collaborations as an implementation tool--both by internal testing of new initiatives aimed at implementation of research findings into day-to-day practice, and as the subject of a number of ongoing external evaluations commissioned by the NIHR Service Delivery and Organisation (SDO) programme [7]. These internal and external evaluations are supposed to contribute to the evidence base on the efficiency and effectiveness of collaboration and other strategies aimed at increasing applied health research use in multiple populations and settings. However, the process of evaluating the CLAHRC processes and outcomes is still at the initial stage, which explains why so little empirical research on the CLAHRCs has been published so far. This paper will explore an alternative way of looking at the subject; it will attempt to draw some lessons for the CLAHRCs by analysing healthcare collaboration through the lens of CoPs. In doing so, it will refer to the seminal works on CoPs published by Etienne Wenger [2,8,9], as well as empirical studies that have used CoPs in healthcare settings, either as an analytical approach or as a knowledge management tool.

### Communities of practice

A community of practice (CoP) is 'a group of people who share a concern, a set of problems, or a passion about a particular topic, and who deepen their understanding and knowledge of this area by interacting on an ongoing basis' [9]. CoPs can range in size; they can be long or short lived, co-located or distributed, homogeneous or heterogeneous, spontaneous or intentional, unrecognised or institutionalised. Organisations can in turn be interpreted as the 'communities of communities' [10], or 'constellations of interconnected CoPs' [8].

Wenger formulates three defining characteristics of CoPs. First, CoP members interact with one another, establishing relationships and negotiating meaning of their actions through *mutual engagement*. Second, members are bound together by an understanding of a sense of *joint enterprise*, which entails a common set of tasks that CoP members can influence. Finally, they produce over time a *shared repertoire *of routines, words, tools, stories, symbols, or concepts that become part of the CoP practice [8]. Iverson and McFee [11] argue that mutual engagement, joint enterprise, and shared repertoire can be used to determine the existence of CoPs, distinguish between different CoPs, and evaluate communicative processes in them. However, in his later work Wenger reformulates these characteristics and presents the 'structural model' of CoPs consisting of three fundamental elements: a *domain *of knowledge, which defines a set of issues; a *community *of people who care about this domain; and the shared *practice *that they are developing to be effective in their domain [9].

According to Wenger *et al. *[9], CoPs can be distinguished from formal departments, project teams, communities of interest and informal networks along the following five dimensions:

1. Purpose: to create, expand and exchange knowledge, and to develop individual capabilities;

2. Membership: self-selection based on expertise or passion for the topic;

3. Boundaries: fuzzy;

4. What holds them together: passion, commitment, and identification with the group and its expertise;

5. Life cycle: CoPs evolve and end organically; they last as long as there is relevance to the topic and interest in learning together.

This distinction has, however, been criticised for being rather vague and contradictory; for instance, the notion of self-selection contradicts the generally accepted premise that people from the same discipline automatically belong to the same CoP [12]. It also does not take into account the possibility that, under certain conditions, a project team may develop CoP characteristics and hence become a 'true' CoP [13]. This has led some authors to suggest that the CoP is actually an umbrella term for a number of different organisational groupings that are characterised by the support for formal and informal interaction between novices and experts, the emphasis on learning and sharing knowledge, and the investment to foster the sense of belonging among members [12,13]. As shown by Li *et al. *[14] in their systematic review, this rather loose interpretation of the CoP concept gets reflected in the empirical research reports, with the examples of CoPs including informal learning groups, clinical placements, and healthcare collaboratives.

### Applying the CoP approach to the CLAHRCs

The structure, content, and narrative of the paper will be underpinned by the critical realist epistemological approach adopted by the authors. First, because this approach suggests that there is an interdependence, rather than distinction, between 'the theoretical' and 'the empirical' [15,16], the paper will look both at the seminal theoretical writings on CoPs and relevant empirical applications of the theory. Second, because critical realism admits some form of theoretical eclecticism [16,17], the paper will also be referring to other theoretical traditions that have developed outside the CoP approach but are compatible with its premises (*e.g*., literature on professionalism, sociology of science, and knowledge transfer). Finally, because the focus of the realist epistemology is 'generative mechanisms' producing outcomes in certain contexts [18,19], the paper will mainly concentrate on the processes taking place in CoPs, rather than reflect on the definitional clarity of the concept or lack thereof.

It should also be noted that the analysis of Wenger's germinal works shows that the concept and theory of CoPs is still evolving. Originating as a mid-level analytical tool of the theory of social learning that embraces community, identity, meaning, and practice, it later was seen as a technique deliberately used by managers to improve knowledge transfer and organisational performance. This resulted in the development of two different perspectives on CoPs: analytical, using CoPs as a theoretical heuristic to analyse practice; and instrumental, used with an intention to cultivate CoPs or utilise them in order to achieve desirable aims. Although there is an inherent tension between these perspectives, the paper will be referring to both of them: first, because the instrumental approach to CoPs is a natural consequence of their use as a theoretical concept, for all social science theories inevitably have practical implications, especially in the field of implementation research [20] and, second, because this combination may be particularly productive both for research and practice [21].

In line with the two main perspectives outlined above, CoPs are being increasingly used both as a theoretical approach to analyse healthcare organisations and as a practical tool enabling collaborative learning and knowledge mobilisation. The CoP approach has previously been applied to the analysis of healthcare collaboration on at least one occasion. Bate and Robert [22] showed that NHS quality improvement collaboratives, which are aimed at closing the gap between potential and actual performance by testing and implementing changes quickly across many organisations [23], are likely to remain time-limited projects unable to achieve sustainable organisational change unless linked and active CoPs are formed within them. The paper develops this idea by providing a brief review of theoretical and empirical literature on CoPs that is organised around the following three themes: knowledge sharing within and across CoPs; CoP formation and manageability; and CoP identity building. It will attempt to analyse the processes of joint working within the CLAHRCs using the CoP theory, identify potential problems that may arise in the process of healthcare collaboration, and reflect on the possibility of the development of new multiprofessional and multi-organisational CoPs within the CLAHRCs.

## Discussion

### CoP knowledge sharing

The CoP concept emerged within the situated theory of learning that views practice--*i.e*., a domain of collective knowing and doing--as the means through which knowledge dynamics in an organisation unfold. In a socially situated view of learning, individuals continuously combine and modify knowledge through their everyday operations and interactions between each other [24-26]. Apart from explicit, codifiable, 'know-that' knowledge, collective practice generates a great deal of tacit, 'know-how' knowledge, which is embodied in the CoP members' practical skills and expertise [27]. As a result, homogeneous and well-established CoPs create distinct epistemic cultures--*i.e*., cultures that 'create and warrant knowledge,' making up 'how we know what we know' [28]. Knowledge can flow relatively easily within such cultures, whereas it can become sticky at the boundaries between them [29]. The boundaries between CoPs can be classified as syntactic (difference in language), semantic (difference in meaning), and pragmatic (difference in practice), the latter being most difficult to overcome [30,31].

The presence of distinct epistemic cultures and boundaries in healthcare has been demonstrated by previous empirical research. It has shown, for instance, that doctors, nurses, and managers have different attitudes to organisational change that are deeply embedded in their professional cultures [32-35], and that there are multiple differences of values, structures, education, and relationships between the acute and primary care sectors [36]. Professional CoPs in healthcare are predominantly unidisciplinary, tend to seal themselves off from neighbouring professional communities and are highly institutionalised, which facilitates knowledge flow within their boundaries, but causes the 'stickiness' of knowledge across them [37]. The epistemic boundaries are especially problematic when different professions are co-located within multiprofessional organisations. The co-existence of partially incompatible epistemic cultures challenges knowledge sharing that occurs in a context of potential tensions and conflict [38].

These findings have direct implications for the CLAHRCs as large-scale multiprofessional and multi-agency network organisations that bring together members of different, well-established CoPs with clearly demarcated boundaries, distinct and partially incompatible epistemic cultures and, especially in the case of medical professionals, supported by powerful professional organisations. The problem of interaction and knowledge sharing within the CLAHRC teams can potentially occur at multiple points. First, there will be inevitable tensions in the communication between the 'worlds' of researchers and practitioners, who can have difficulties communicating with each other given the differences in their epistemic cultures [39]. Second, it should be noted that both of these worlds are not homogeneous; on the contrary, they are represented by different professional and occupational CoPs. Thus, we could expect tensions between clinical researchers employed by medical schools, whose mode of functioning is largely based on a positivist biomedical paradigm, and the implementation researchers representing social sciences and often adopting more descriptive ethnographic approaches to organisations grounded, for instance, in social constructionism and symbolic interactionism. Similarly, the 'world' of clinicians also consists of multiple professional groupings: doctors, nurses, and allied health professionals, with different medical specialities (*e.g*., surgeons, neurologists, cardiologists) tending to form their own communities, which often spread across organisations, but are still likely to retain their own disciplinary boundaries when co-located in the CLAHRCs with other professions. Finally, there is a boundary between clinical practice and healthcare management--two fields with profound differences in cultures, perceptions of research evidence, and the nature of decision-making processes [40].

To cross these boundaries, the CLAHRCs might utilise the following types of bridges (see also Table 1 for more examples):

**Table 1:** 'Bridges' that could be used by the CLAHRCs to cross the boundaries between CoPs

Type	Examples
Knowledge brokers	Clinical managers Clinicians involved in management Clinicians involved in research Clinicians involved in quality improvement Internal facilitators (*e.g*., knowledge transfer associates) External facilitators (*e.g*., management consultants)
Boundary objects	Dashboards Websites Powerpoint presentations Quality and outcomes framework (QOF) Primary care registers Patient alert cards Clinical pathways and protocols Assessment tools
Boundary interactions	Face-to-face meetings Practice visits Learning sessions Online forums WebEx conferences Focus groups

1. Knowledge brokers--people who, having membership in several CoPs, seek to facilitate interaction and coordinate practice between them. Knowledge brokering is often performed by individuals with hybrid professional roles--for example, clinical managers may span boundaries between the management and the medical professions [41,42].

2. Boundary objects--artifacts, discourses, and processes possessing interpretative flexibility that allows them to overcome syntactic, semantic, and pragmatic boundaries, and hence contribute to knowledge transfer across CoPs [43,44]. For example, x-rays and treatment protocols have been shown to play the role of boundary objects in a multidisciplinary cancer care team [45].

3. Boundary interactions among people from different CoPs--these include single or discrete boundary encounters (*e.g*., meetings, visits, and delegations) and longer-lived practice-based connections, including cross-disciplinary projects [8,24].

Cross-disciplinary projects, including those implemented by the CLAHRCs, can be considered as a variant of boundary practice because participating in this kind of project exposes practitioners to specific tasks going beyond their normal practices and forces them to negotiate their own competences with the competencies of others. While it is possible that some of the CLAHRC multidisciplinary projects may indeed become a bridge between different CoPs (this possibility will be addressed in more detail in the next subsection), the following potential obstacles to knowledge sharing within these projects need to be considered. First, these boundary projects can become CoPs in their own right and develop their own boundaries, which can prevent them from functioning as knowledge brokers between the wider communities these newly formed CoPs had intended to link [46]. Second, as far as the level of individual knowledge brokers is concerned, full participation in one CoP may render brokering difficult: those with multimembership seeking to coordinate across CoPs may have difficulties participating fully as members of one community if they have allegiances in another [24]. Finally, some of the more excessively formalised boundary objects, such as project plans, performance targets, or clinical guidelines can become a barrier to successful collaboration by legitimising interprofessional differences, reinforcing existing power structures, and maintaining occupational control over task areas [25,47].

### CoP formation and manageability

As shown in the previous subsection, one of the ways to enhance knowledge transfer and learning at the boundaries between CoPs is the creation of a cross-disciplinary project that can act as a bridge between CoPs and, under certain conditions, become a multiprofessional CoP in its own right. One of the CLAHRC aims is to 'link those who conduct applied health research with all those who use it in practice across the health community covered by the collaboration' [7]. This may be interpreted as an imperative for creating new multiprofessional and multi-organisational CoPs within the CLAHRC projects, which would bring together representatives of multiple communities. The following subsection will explore the literature concerned with the development of multiprofessional and multi-organisational CoPs in collaborative settings.

The issue of CoP formation has been viewed differently in the seminal CoP literature and remains an area of debate. CoPs, as defined by Lave and Wenger [2], cannot be deliberately designed by managers; an organisation can only establish a team for a particular project, which may later emerge as a CoP [1]. Wenger and Snyder [48] suggest that managers cannot mandate CoPs because the organic, spontaneous, and informal nature of the communities makes them resistant to supervision and interference. At the same time they argue that CoPs may benefit from cultivation by managers, who should identify communities that can potentially enhance the company's strategic capabilities and provide the infrastructure that will support such communities and enable them to apply their expertise effectively. More recent contributions suggest that CoPs can be cultivated intentionally; furthermore, these deliberate communities may be more useful for an organisation than the organic ones [9,49].

Under the influence of the theoretical literature mainly concerned with private sector organisations, deliberately constructed CoPs are getting increasingly used as a knowledge management tool in healthcare. Although the empirical evidence is still limited, it can be concluded that the formation of a genuine multiprofessional CoP is rare but possible [37]. The CoP approach has been demonstrated to enhance interprofessional clinical practice [50], facilitate quality improvement, encourage buy-in among participants, promote knowledge transfer [51], and contribute to the development of services spanning the interests of different stakeholders [52]. The following key factors that influence the development, functioning and maintenance of multiprofessional CoPs have been described: membership--selecting the members, the extent (active or passive) and legitimacy of their involvement; commitment to the desired goals; relevance to local communities and the existing services; enthusiasm; infrastructure to support the work of CoPs; skills in accessing and appraising evidence; and resources [52].

However, it should be emphasised that several crucial questions about the formation of multiprofessional CoPs still remain unanswered. First, it is not clear what organisational, group-level, and individual factors may enhance the transition from a team to a CoP. Second, in spite of the reports describing the deliberate formation of 'genuine' multiprofessional CoPs from scratch, their description is often not informative enough to judge to what extent these groupings differ from the project teams in terms of achieving mutual engagement, joint enterprise, and shared repertoire. It may well be that the groupings that are labelled as CoPs represent a rhetorical device rather than organic CoPs characterised by shared practice and sense of belonging. Finally, we do not know whether and how horizontal, informal, egalitarian multiprofessional communities can emerge and function in a context where they have to co-exist with the vertical, formal, command-and-control structures of the NHS, given the evidence suggesting that the excessive legitimisation and formalisation of 'organic' CoPs can disrupt, rather than support, their knowledge-sharing capacity [53,54].

Compared to the previous collaboration initiatives in the NHS, the CLAHRCs are characterised by the more voluntary nature of involvement, placement in the context of local healthcare needs, and an emphasis on capacity building and learning. These factors may increase the probability of supplying newly formed multiprofessional projects with enthusiastic members who will be committed to achieving the relevant goals, thus addressing some of the factors mentioned above as prerequisites for the formation of multiprofessional CoPs. It has also been suggested that the CLAHRC being co-funded by the NIHR and the NHS Trusts will result in a collaborative model of ownership with a broad range of stakeholders having a vested interest in shaping the strategic direction of the collaboration [55]. It is, however, unclear whether this will lead to the formation of CoPs, given the potentially conflicting partners' agendas, the continuous process of NHS reform distracting organisational resources from joint working and, most importantly, the dynamic membership within the CLAHRCs, given the potential for NHS trusts to opt out of the collaboration should their priorities change.

Whether formed organically within the CLAHRC cross-disciplinary projects or deliberately cultivated by the CLAHRC management, the multiprofessional CoPs will have to be maintained, directed, and controlled to achieve desired aims. Paradoxically, while it has been suggested that managers play a critical role in constructing, aligning, and supporting CoPs [9,49], there is little empirical evidence for these assertions. Furthermore, it has been argued that managers are incapable of making the CoP a direct instrument of policy and control [56]. In spite of facilitation, the knowledge transfer in these communities does not necessarily follow the model of evidence-based practice, but is shaped strongly by the personal, political, and professional agendas of the participants [57]. It can thus be concluded that the deliberate cultivation of multiprofessional CoPs within the CLAHRCs might help to solve the problems of knowledge sharing outlined in the previous subsection, but the extent to which these CoPs can be constructed and directed remains unclear.

### CoP identity building

The concept of identity building occupies one of the central places in the CoP approach that emphasises that the negotiation of a common identity is a prerequisite for forming a community. Wenger [8] suggests the following characterisations of identity:

1. Identity as negotiated experience: participation in a CoP and (often unspoken) negotiating the meanings of this experience with other CoP members;

2. Identity as community membership: translation of the CoP membership into an identity as a form of competence;

3. Identity as a learning trajectory: a coherent process of changing forms of participation within a CoP over time;

4. Identity as nexus of multimembership: an experience of multimembership in various CoPs and reconciliation of different identities to maintain one identity across boundaries;

5. Identity as a relation between the local and the global: negotiating local ways of belonging to broader constellations of CoPs.

Regardless of whether there is an explicit intention to cultivate CoPs within the CLAHRC or any other collaboration, the multiprofessional and multi-agency environment in which these projects are located mandates an understanding how a new 'collaborative' identity is negotiated by the participants. It should be noted, however, that the processes of identity formation in multiprofessional and multi-agency CoPs are not specifically addressed in the seminal CoP literature. To discuss potential problems related to the formation of the 'collaborative' identities within the CLAHRC, it is helpful to refer to the concepts of professional and organisational identification.

Professional identity can be defined as 'the relatively stable and enduring constellation of attributes, beliefs, values, motives, and experiences in terms of which people define themselves in a professional role' [58]. Though professional identity is not static, there seems to be a core identity that remains stable and with which all members are able to identify. Around this lies an extended identity that is subject to modification as the result of widening fields of work, increased knowledge and skills, changes in attitudes and values, or re-interpretation of old ones [59]. It should also be emphasised that the development of professional identity in healthcare professions should be analysed in relation to the concepts of professional dominance, collegiality and autonomy [60].

Several empirical studies are worth mentioning in this respect. In a qualitative study on interagency and interprofessional teams in the NHS, Robinson and Cottrell [61] argue that in multiprofessional work, professional knowledge boundaries can become blurred and professional identity can be challenged as roles and responsibilities change. As a result, team members may struggle to cope with the disintegration of one version of professional identity before a new version can be built. Baxter and Brumfitt [62] examine interprofessional practice in multidisciplinary stroke care and conclude that the depth of professional knowledge and skills is perceived as the core element in preserving professional differences; that although some role substitution is possible, there is little evidence of role boundary blurring between professions, and that there is variation among staff whether they consider themselves first as a member of a particular profession, or mainly as a member of a local team. Finally, in their study of a multiprofessional radiotherapy unit, Tagliaventi and Mattarelli [26] emphasise the importance of flexibility of professional identities as a facilitator of knowledge transfer at the boundaries of different communities, when CoP members, faced with the need to cooperate, temporarily suspend their community identity in order to capture the languages and actions proper to the members of other communities.

This has a number of implications for the CLAHRCs. First, building a new, shared collaborative identity may be complicated by the perceived status differentials between the professional groups, with medical doctors traditionally possessing more power, autonomy, and control. Second, professional collegiality, when members of a profession have similar perceptions, values, and experiences, may lead to the prevalence of traditional uniprofessional CoPs over new multiprofessional communities. Third, it is not clear to what extent those CLAHRC participants who work at the boundaries between uniprofessional communities will be able to effectively extend their professional identities and adjust to their new hybrid, boundary-spanning roles. Finally, at the individual level, it can be anticipated that committed participation in cross-disciplinary CLAHRC projects might result in internal psychological conflicts, which may be represented by the stressful disintegration of professional identity, as well as in tensions with those colleagues who still operate in traditional uniprofessional communities.

Because the CLAHRCs involve people who are at the same time employed by other organisations, such as universities, primary care trusts, and acute trusts, the analysis of identity formation within the CLAHRC cross-disciplinary projects should also consider the notion of organisational identification. This is defined as a 'form of psychological attachment that occurs when members adopt the defining characteristics of the organisation as defining characteristics for themselves' [63]. A member's level of organisational identification indicates the degree to which his/her membership in an organisation is tied to the content of his/her self-concept [64]. Organisational identification is most likely to occur under conditions where the boundaries between one's own organisation and other organisations are salient, when membership in the organisation is attractive, and when organisational categories best account for similarities and differences across individuals and groups [65].

Organisations forming the CLAHRCs may have differing organisational cultures as well as potentially conflicting motivations to collaborate and different interpretations of the process of collaboration itself. It can thus be expected that the identification with the CLAHRCs may be impeded by them being heterogeneous, temporary, network-type organisations without clear boundaries or a distinctive organisational image. Reconciliation of multiple organisational identities of the CLAHRC participants with a new collaborative identity, which is necessary for the attainment of a shared vision, could thus prove difficult. Finally, the functional separation of research and implementation strands, which occurs in some CLAHRCs, may limit the opportunities for sharing practice, negotiating new identities, and knowledge transfer between the communities of researchers and practitioners.

## Summary

### CLAHRCs and CoPs: the analytical perspective

The CoP approach can be considered a mid-range theory analysing the processes of joint working, identity building, and knowledge sharing as a function of smaller, sub-organisational groupings that are distinguished by shared practices, meanings, and epistemic cultures. Focusing on the issues related to learning, meaning, and identity within and across those groupings, it provides an insightful analytical approach that can complement more traditional, rationalistic, 'stage-of-change' theories used in the field of implementation research [66]. The main strength of the CoP theory is that it is able to provide a basis for the development and delivery of theory-informed implementation interventions as well as their evaluations, which is especially important in the current situation when theory is not sufficiently utilised in the field of implementation research [67].

All these factors make CoP a useful lens for looking at healthcare collaboration and analysing the range of issues that may be faced by initiatives such as CLAHRCs in the process of their interprofessional and inter-organisational work. Although the CLAHRCs vary in form and focus, they have to deal with the same set of major objectives, which can hardly be achieved without promoting effective collaboration between various groups of stakeholders. As highlighted by a recent external evaluation of the CLAHRC for Leicestershire, Northamptonshire and Rutland, a special emphasis should be placed on the incorporation of social science and management sciences into the CLAHRC projects, encouraging interdisciplinary learning within the CLAHRC and developing a more integrated partnership for the operation of the CLAHRC [68]. As the paper has attempted to demonstrate, the CoP approach may be a useful heuristic for understanding and informing these processes.

### CLAHRCs and CoPs: the instrumental perspective

Like any other healthcare partnerships and collaboratives, the CLAHRCs have to co-exist and integrate with a constellation of various well-established, mainly uniprofessional, communities of researchers, doctors, nurses, managers, and other healthcare professionals. These communities have distinct and partially incompatible epistemic cultures, which leads to the formation of multiple semantic, syntactic, and pragmatic boundaries hampering the process of joint working. Not only can the CoP approach illuminate these boundaries; it can also suggest ways of crossing them to enable knowledge transfer and organisational learning. There may exist multiple ways of influencing the CoPs involved in the CLAHRC projects that still need to be assessed and evaluated by empirical research. This paper will therefore avoid providing prescriptive solutions to the problem of joint working within the CLAHRC. Instead, it suggests a brief list of questions to be addressed when designing interventions and evaluations informed by the CoP theory (See Table 2).

**Table 2:** Issues to be addressed by the collaborative projects informed by the CoP theory

Area	Questions to be considered
Knowledge sharing between existing CoPs involved in a multiprofessional/multi-organisational project	• What are the main CoPs involved in a project? • What are the boundaries between those communities? • What are the existing communication patterns within and across those communities? • What potential knowledge brokers can be involved in the project? • What boundary objects might be used to link separated CoPs? • What boundary interactions between the CoPs can be facilitated by the project?
Development of new interdisciplinary and inter-organisational communities of practice	• What is being done to promote the formation of new boundary practices centred around the activities of the collaboration? • What are the existing networks that the project can link to? • Is building a community recognised as a priority by the management of the project? • What is being done to make the boundaries of the new community permeable and promote knowledge transfer to other settings?
Developing sufficient identification with the Collaboration	• What is the distribution of power between the key individuals and communities involved in the project? • Does the project create a positive image that may persuade professionals to join it and work constructively in a collaborative way? • How is the development of functional flexibility and hybrid professional roles supported by the project? • How does the project satisfy expectations, agendas and motivations of different parties involved? • How can potential problems relating to multimembership in several communities be envisaged and prevented?

The CLAHRCs have been charged with an ambitious goal of creating a new, distributed model for the conduct and application of applied health research that links producers and users of research. It could be hypothesised that producing a sustainable change in the ways applied health research is conducted and implemented might require the cultivation of new multiprofessional and multi-organisational CoPs within the CLAHRCs, united by shared practice and a shared sense of belonging. However, the formation of these communities may be hampered by unfavourable contextual factors, while participants' identification with the collaborations may be influenced by issues related to professional power, autonomy, and collegiality--as well as their commitment to the institutions from which they originate. In addition, the evidence on the existence and effects of such CoPs remains sketchy; even if active and effective CoPs, whether organic or deliberately cultivated, develop within the CLAHRCs, their manageability is likely to remain limited.

### Analytical and theoretical perspectives: integration and future research

The literature deploying the instrumental perspective on CoPs and concerned with their 'cultivation,' tends to take the very possibility of deliberate creation of such communities for granted. It mainly focuses on the advantages of using this approach, but does not seem to provide sufficient explanation of how these newly formed CoPs develop, what characteristics they possess, and how they interact with a wider organisational context. To address these issues, further empirical research is required, based on the combination of the analytical and instrumental perspectives on CoPs outlined above. This complex perspective may provide more insight in the processes taking place in CoPs that have been deliberately cultivated for enhancing knowledge exchange, learning, and innovation. It may also help to identify key differences between the 'organic' and 'deliberate' CoPs, and answer the question of whether we should attempt to cultivate new CoPs or focus on fostering a better relationship between the existing organic ones instead.

It should also be emphasised that both theoretical and empirical CoP literature has mainly focused on uniprofessional CoPs. As a result, the development, functioning, and effects of multiprofessional and multi-agency CoPs, be they organic or cultivated, remains an underresearched area. Further research is required to identify contextual factors that can facilitate their formation, describe the dynamics of actors' interactions within these communities, and analyse how the members reconcile their existing professional and organisational identities with a new 'collaborative' identity. Crossing inter-organisational boundaries and bringing together people from different professional backgrounds within the relatively long life span of the initiative make the CLAHRCs an optimal setting for studying multi-organisational and multiprofessional CoPs.

## Competing interests

RK is a recipient of the CLAHRC PhD studentship, GH is an Academic Lead for the Greater Manchester CLAHRC, and KW is Director of the NIHR SDO programme, but they write here in a personal capacity. The views and opinions expressed therein are those of the authors and do not necessarily reflect those of the NIHR SDO programme or the CLAHRCs.

## Authors' contributions

All authors contributed to the conception and design of the paper. RK reviewed the literature and drafted the manuscript. GH and KW revised the manuscript. All authors read and approved the final manuscript.

## References

[B1] Roberts J Limits to communities of practice Journal of Management Studies 2006 43 623 639 10.1111/j.1467-6486.2006.00618.x

[B2] Lave J Wenger E Situated Learning: Legitimate Peripheral Participation 1991 Cambridge: Cambridge University Press

[B3] le May A Communities of Practice in Health and Social Care 2009 Oxford: Wiley-Blackwell

[B4] Collaborations for Leadership in Applied Health Research and Care http://www.nihr.ac.uk/files/pdfs/CLAHRC%20-%20Call%20for%20Proposals%20for%20Pilots.pdf

[B5] NIHR Collaborations for Leadership in Applied Health Research and Care (CLAHRCs) http://www.nihr.ac.uk/infrastructure/Pages/infrastructure_clahrcs.aspx 10.1161/STROKEAHA.113.003216 24385272

[B6] Cooksey D A review of UK health research funding 2006 London: The Stationery Office

[B7] Evaluating Partnerships between Universities and NHS Organisations: Learning from the NIHR Collaborations for Leadership in Applied Health Research and Care (CLAHRC) http://www.sdo.nihr.ac.uk/files/researchcall/1075-brief.pdf

[B8] Wenger E Communities of practice: learning, meaning and identity 1998 Cambridge: Cambridge University Press

[B9] Wenger E McDermott RA Snyder W Cultivating communities of practice: a guide to managing knowledge 2002 Boston, MA: Harvard Business Press

[B10] Brown JS Duguid P Organizational Learning and Communities-of-Practice: Toward a Unified View of Working, Learning and Innovation Organization Science 1991 2 40 57 10.1287/orsc.2.1.40

[B11] Iverson JO McPhee RD Communicating Knowing through Communities of Practice: Exploring Internal Communicative Processes and Differences among CoPs Journal of Applied Communication Research 2008 36 176 199 10.1080/00909880801923738

[B12] Li LC Grimshaw JM Nielsen C Judd M Coyte PC Graham ID Evolution of Wenger's concept of community of practice Implementation Science 2009 4 11 10.1186/1748-5908-4-11 19250556 PMC2654669

[B13] Hildreth PM Going virtual: distributed communities of practice 2004 London: Idea Group Publishing

[B14] Li LC Grimshaw JM Nielsen C Judd M Coyte PC Graham ID Use of communities of practice in business and health care sectors: A systematic review Implementation Science 2009 4 27 10.1186/1748-5908-4-27 19445723 PMC2694761

[B15] Hands DW Reflection without Rules: Economic Methodology and Contemporary Science Theory 2001 Cambridge: Cambridge University Place

[B16] Sayer A Method in Social Science: A Realist Approach 1992 2 London: Routledge

[B17] Danermark B Ekström M Jakobsen L Karlsson J Explaining Society: Critical Realism in the Social Sciences 2002 Oxon: Routledge

[B18] Pawson R Tilley N Realistic Evaluation 1997 London: SAGE Publications

[B19] Sayer A Realism and Social Science 2000 London: SAGE Publications

[B20] Eccles M Grimshaw J Walker A Johnston M Pitts N Changing the behavior of healthcare professionals: the use of theory in promoting the uptake of research findings Journal of Clinical Epidemiology 2005 58 107 112 10.1016/j.jclinepi.2004.09.002 15680740

[B21] Wenger E Blackmore C Communities of practice and social learning systems: the career of a concept Social Learning Systems and Communities of Practice 2010 London: Springer

[B22] Bate SP Robert G Knowledge management and communities of practice in the private sector: Lessons for modernizing the National Health Service in England and Wales Public Administration 2002 80 643 663 10.1111/1467-9299.00322

[B23] Øvretveit J Bate P Cleary P Cretin S Gustafson D McInnes K McLeod H Molfenter T Plsek P Robert G Shortell S Wilson T Quality collaboratives: lessons from research Quality and Safety in Health Care 2002 11 345 351 10.1136/qhc.11.4.345 12468695 PMC1757995

[B24] Wenger E Communities of practice and social learning systems Organization 2000 7 225 246 10.1177/135050840072002

[B25] Bechky BA Sharing Meaning across Occupational Communities: The Transformation of Understanding on a Production Floor Organization Science 2003 14 312 330 10.1287/orsc.14.3.312.15162

[B26] Tagliaventi MR Mattarelli E The role of networks of practice, value sharing, and operational proximity in knowledge flows between professional groups Human Relations 2006 59 291 319 10.1177/0018726706064175

[B27] Brown JS Duguid P Knowledge and Organization: A Social-Practice Perspective Organization Science 2001 12 198 213 10.1287/orsc.12.2.198.10116

[B28] Knorr-Cetina K Epistemic Cultures: How the Sciences Make Knowledge 2000 Cambridge, MA: Harvard University Press

[B29] Duguid P 'The art of knowing': social and tacit dimensions of knowledge and the limits of the community of practice The Information Society 2005 21 109 118 10.1080/01972240590925311

[B30] Carlile PR A pragmatic view of knowledge and boundaries: Boundary objects in new product development Organization Science 2002 13 442 455 10.1287/orsc.13.4.442.2953

[B31] Carlile PR Transferring, translating, and transforming: An integrative framework for managing knowledge across boundaries Organization Science 2004 15 555 568 10.1287/orsc.1040.0094

[B32] Degeling P Kennedy J Hill M Mediating the cultural boundaries between medicine, nursing and management - the central challenge in hospital reform Health Serv Manage Res 2001 14 36 48 10.1258/0951484011912519 11246783

[B33] Degeling P Maxwell S Kennedy J Coyle B Medicine, management, and modernisation: a 'danse macabre'? BMJ 2003 326 649 652 10.1136/bmj.326.7390.649 12649244 PMC1125544

[B34] Hall P Interprofessional teamwork: Professional cultures as barriers Journal of Interprofessional Care 2005 19 188 196 10.1080/13561820500081745 16096155

[B35] Morgan PI Ogbonna E Subcultural dynamics in transformation: A multi-perspective study of healthcare professionals Human Relations 2008 61 39 65 10.1177/0018726707085945

[B36] Fitzgerald L Ferlie E Wood M Hawkins C Interlocking Interactions: The Diffusion of Innovations in Health Care Human Relations 2002 55 1429 1449 10.1177/001872602128782213

[B37] Ferlie E Fitzgerald L Wood M Hawkins C The nonspread of innovations: The mediating role of professionals The Academy of Management Journal 2005 48 117 134

[B38] Mørk BE Aanestad M Hanseth O Grisot M Conflicting epistemic cultures and obstacles for learning across communities of practice Knowledge and Process Management 2008 15 12 23 10.1002/kpm.295

[B39] Nutley S Davies HTO Making a Reality of Evidence-Based Practice: Some Lessons from the Diffusion of Innovations Public Money & Management 2000 20 35 4221696874

[B40] Walshe K Rundall TG Evidence-based Management: From Theory to Practice in Health Care Milbank Quarterly 2001 79 429 457 10.1111/1468-0009.00214 11565163 PMC2751196

[B41] Fitzgerald L Ferlie E Professionals: Back to the future? Human Relations 2000 53 713 739 10.1177/0018726700535005

[B42] Lorbiecki A Soothill K, Mackay, L, Webb K Clinicians as managers: Convergence or collision? Interprofessional Relations in Health Care 1995 London: Edward Arnold

[B43] Star SL Griesemer JR Institutional ecology, 'translations' and boundary objects: Amateurs and professionals in Berkeley's Museum of Vertebrate Zoology, 1907-39 Social Studies of Science 1989 19 387 420 10.1177/030631289019003001

[B44] Swan J Bresnen M Newell S Robertson M The object of knowledge: The role of objects in biomedical innovation Human Relations 2007 60 1809 1837 10.1177/0018726707084915

[B45] Oborn E Dawson S Learning across Communities of Practice: An Examination of Multidisciplinary Work British Journal of Management 2010 21 843 858 10.1111/j.1467-8551.2009.00684.x

[B46] Bullough RV Draper RJ Smith L Birrell JR Moving beyond collusion: Clinical faculty and university/public school partnership Teaching and Teacher Education 2004 20 505 521 10.1016/j.tate.2004.04.007

[B47] Oswick C Robertson M Boundary objects reconsidered: from bridges and anchors to barricades and mazes Journal of Change Management 2009 9 179 193 10.1080/14697010902879137

[B48] Wenger EC Snyder WM Communities of practice: The organizational frontier Harvard Business Review 2000 78 139 14611184968

[B49] Saint-Onge H Wallace D Leveraging communities of practice for strategic advantage 2003 Burlington, MA: Butterworth-Heinemann

[B50] White D Suter E Parboosingh IJ Taylor E Communities of practice: Creating opportunities to enhance quality of care and safe practices Healthcare Quarterly 2008 11 80 84 18382166 10.12927/hcq.2008.19654

[B51] Bentley C Browman GP Poole B Conceptual and practical challenges for implementing the communities of practice model on a national scale--a Canadian cancer control initiative BMC Health Services Research 2010 10 3 10.1186/1472-6963-10-3 20051125 PMC2820037

[B52] Lathlean J le May A Communities of practice: An opportunity for interagency working Journal of Clinical Nursing 2002 11 394 398 10.1046/j.1365-2702.2002.00630.x 12010537

[B53] Addicott R McGivern G Ferlie E Networks, Organizational Learning and Knowledge Management: NHS Cancer Networks Public Money & Management 2006 26 87 94 10.1111/j.1467-9302.2006.00506.x21696874

[B54] Addicott R McGivern G Ferlie E The Distortion of a Managerial Technique? The Case of Clinical Networks in UK Health Care British Journal of Management 2007 18 93 105 10.1111/j.1467-8551.2006.00494.x

[B55] Gerrish K Tapping the potential of the National Institute for Health Research Collaborations for Leadership in Applied Health Research and Care (CLAHRC) to develop research capacity and capability in nursing Journal of Research in Nursing 2010 15 215 225 10.1177/1744987110363214

[B56] Swan J Scarbrough H Robertson M The construction of 'communities of practice' in the management of innovation Management Learning 2002 33 477 496 10.1177/1350507602334005

[B57] Gabbay J le May A Jefferson H Webb D Lovelock R Powell J Lathlean J A case study of knowledge management in multiagency consumer-informed 'communities of practice': Implications for evidence-based policy development in health and social services Health (London) 2003 7 283 310 10.1177/1363459303007003003

[B58] Ibarra H Provisional Selves: Experimenting with Image and Identity in Professional Adaptation Administrative Science Quarterly 1999 44 764 791 10.2307/2667055

[B59] Hornby S Atkins J Collaborative care: interprofessional, interagency and interpersonal 2000 2 Oxford: Blackwell Science

[B60] Hudson B Interprofessionality in health and social care: the Achilles' heel of partnership? Journal of Interprofessional Care 2002 16 7 17 10.1080/13561820220104122 11915720

[B61] Robinson M Cottrell D Health professionals in multi-disciplinary and multi-agency teams: Changing professional practice Journal of Interprofessional Care 2005 19 547 560 10.1080/13561820500396960 16373211

[B62] Baxter SK Brumfitt SM Professional differences in interprofessional working Journal of Interprofessional Care 2008 22 239 251 10.1080/13561820802054655 18569411

[B63] Dutton JE Dukerich JM Harquail CV Organizational images and member identification Administrative Science Quarterly 1994 39 239 263 10.2307/2393235

[B64] Dukerich JM Golden BR Shortell SM Beauty is in the eye of the beholder: the impact of organizational identification, identity, and image on the cooperative behaviors of physicians Administrative Science Quarterly 2002 47 507 537 10.2307/3094849

[B65] Pratt MG Whetten DA, Godfrey PC To be or not to be? Central questions in organizational identification Identity in Organizations: Building Theory through Conversations 1998 London: SAGE Publications

[B66] Grol RP Bosch MC Hulscher ME Eccles MP Wensing M Planning and Studying Improvement in Patient Care: The Use of Theoretical Perspectives Milbank Quarterly 2007 85 93 138 10.1111/j.1468-0009.2007.00478.x 17319808 PMC2690312

[B67] The Improved Clinical Effectiveness through Behavioural Research Group (ICEBeRG) Designing theoretically-informed implementation interventions Implement Sci 1 4 10.1186/1748-5908-1-4 PMC1436012 16722571

[B68] Øvretveit J Lomas J Davies H Powell A External Advisory Review of the LNR CLAHRC: report to the management board 2010

